# Imaging findings and clinical features of atypical retroperitoneal abscess caused by duodenal perforation: a case report and review of the literature

**DOI:** 10.1186/s13256-020-02393-x

**Published:** 2020-07-17

**Authors:** Xijin Mao, Ning Yu, Xingfang Jia, Wanfeng Fan

**Affiliations:** 1 Department of Radiology, Binzhou Medical University Hospital, Binzhou, 256603 Shandong China; 2 Department of Pathology, Binzhou Medical University Hospital, Binzhou, 256603 Shandong China; 3 Department of Gastroenterology, Binzhou Medical University Hospital, Binzhou, 256603 Shandong China

**Keywords:** Atypical retroperitoneal abscess, Duodenal perforation, Computed tomography

## Abstract

**Introduction:**

A retroperitoneal abscess caused by duodenal perforation is a relatively rare disease clinically. We report the case of a patient with a local high-density shadow at the head of the retroperitoneal pancreas.

**Case presentation:**

A 28-year-old Chinese man presented with fever and abdominal pain after overeating and heavy drinking. On physical examination, he had mild tenderness in his upper abdomen. Laboratory examination results showed a white blood cell count of 24.06 10^9^/L and a neutrophil absolute value of 18.81 10^9^/L, and a computed tomography scan showed an irregular soft tissue mass with uneven enhancement of the cystic wall in the retroperitoneal space. Gastroscopy showed that there was a fistula in the anterior wall of the duodenal bulb. Endoscopic anastomosis clip system (over-the-scope clip) of the duodenal fistula was performed successfully. After the operation, nasal feeding was provided with a nutrition tube, and empiric anti-infection, acid-inhibiting, and stomach-protecting treatments were administered. Our patient’s body temperature gradually returned to normal, and his abdominal pain decreased.

**Conclusions:**

A retroperitoneal abscess caused by duodenal perforation can be diagnosed by clinical symptoms and abdominal computed tomography imaging. The choice of treatment should be based on accurate and timely clinical and imaging data.

## Introduction

Clinically, retroperitoneal abscesses due to duodenal perforation are relatively rare. Altemeier [1] reported that two out of 189 cases of retroperitoneal abscess were caused by duodenal perforation, a proportion of only 0.95%. Duodenal perforation is often associated with peptic ulcer disease (duodenal ulcer), iatrogenic causes, and trauma [2–4], and duodenal perforation will eventually occur in 2–10% of patients with duodenal ulcers [3]. Major causes of peptic ulceration and perforation include *Helicobacter pylori* infection and non-steroidal anti-inflammatory drugs (NSAIDs) [2]. In contrast, duodenal perforation caused by trauma is relatively rare, and less than 2% of all abdominal injuries lead to the condition [5]. The patient in this case came to the hospital because of fever and abdominal pain, and subsequent computed tomography (CT) investigation led to the detection of local high-density shadows in the head of the retroperitoneal pancreas.

## Case presentation

**Fig. 1:**
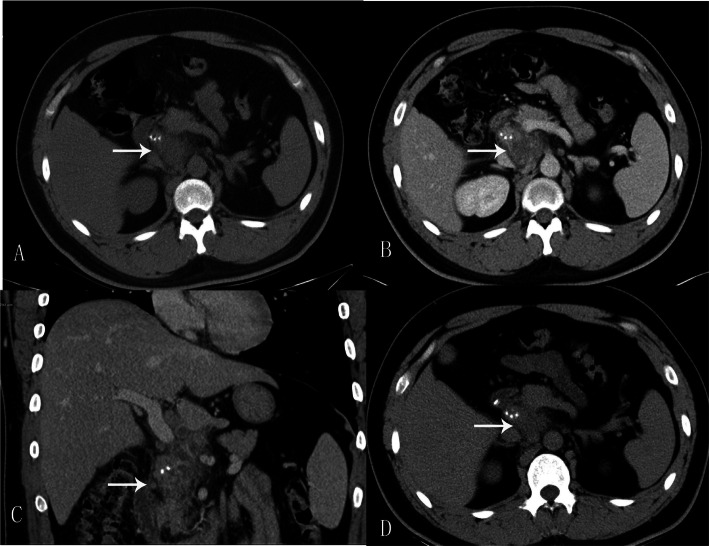
**a** Computed tomography scan without contrast showing irregular soft tissue mass (*White arrow*). The lesion had an uneven interior and was surrounded by a blurred fat gap. There were multiple spots of high density with clear margins in the upper right of the lesion. **b**-**c** Contrast-enhanced computed tomography showing multilocular changes of the lesion (*White arrow*), uneven enhancement of cystic wall, slight enhancement of adjacent duodenal wall, and multiple enlarged lymph nodes. **d** Abdominal computed tomography was reviewed 1 month after treatment. Computed tomography scan without contrast showing the postoperative change of local high-density imaging in the duodenum wall, the volume of the lesion (*White arrow*) gradually decreased, and the edge was clear, and no substantial changes in the multiple high-density shadow spots were seen

**Fig. 2:**
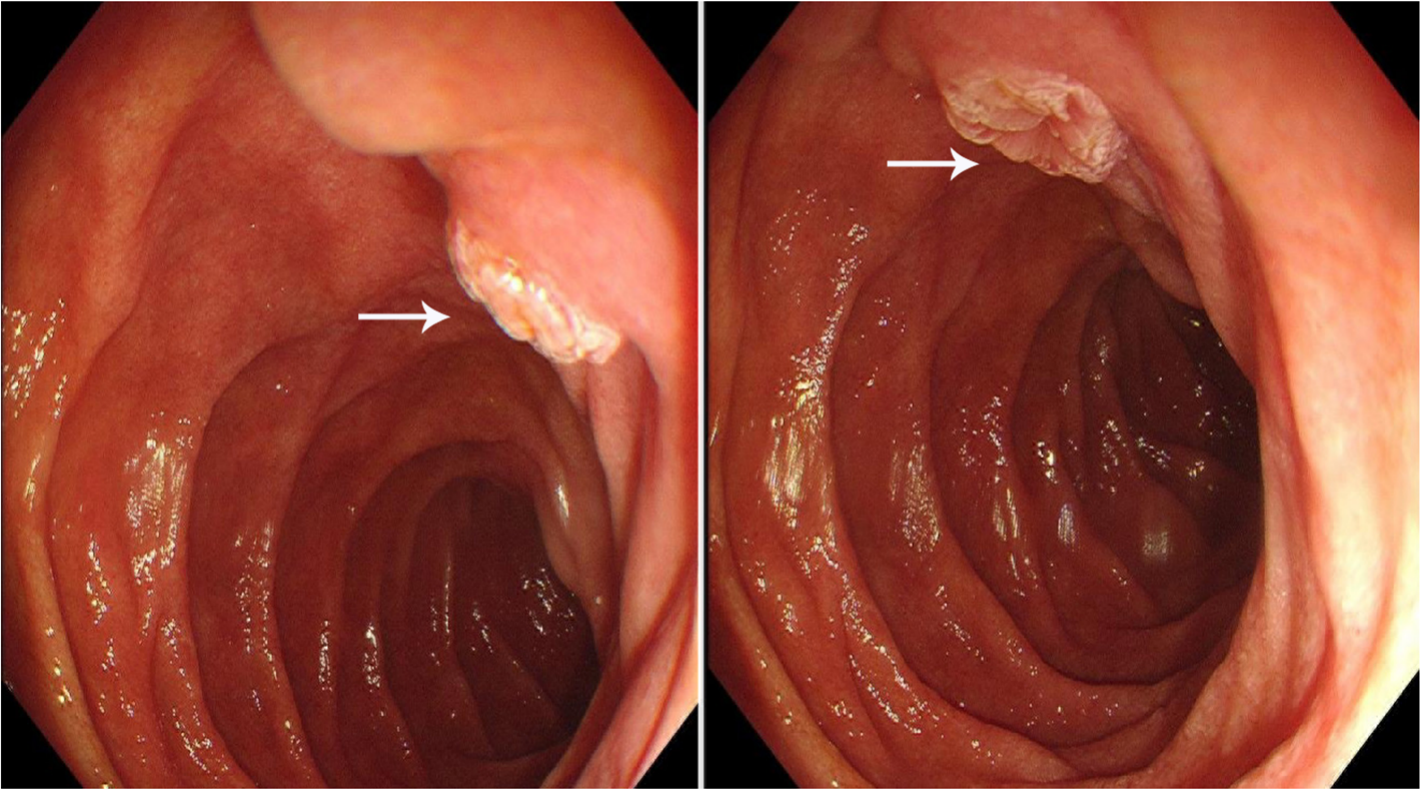
Gastroscopy showing a deep fistula (*White arrow*), about 0.3 cm in size, in the anterior wall of the duodenal bulb with congestion and edema of the surrounding mucosa

**Fig. 3:**
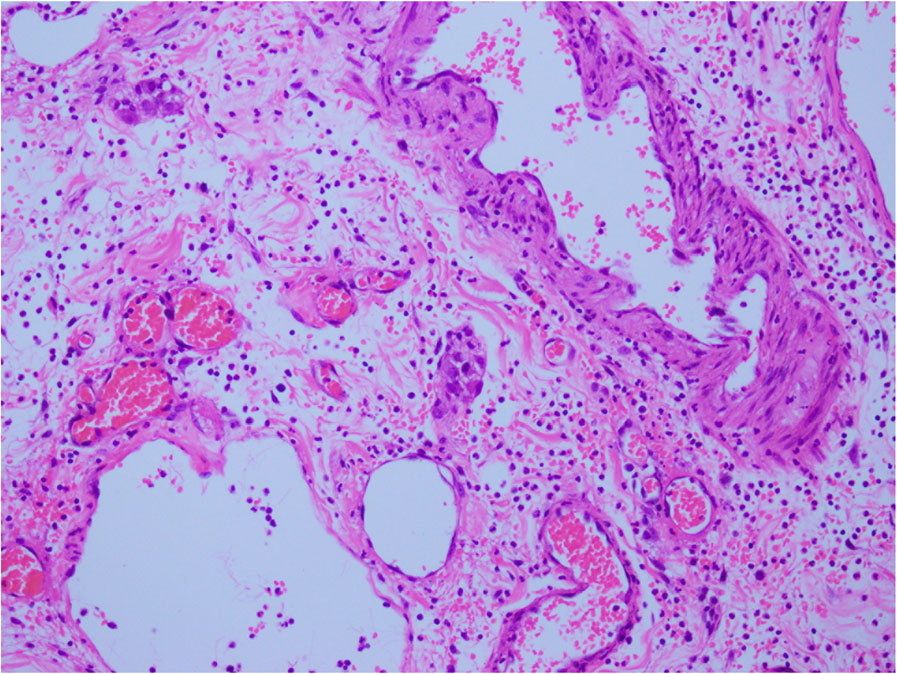
Pathological examination (HE, original magnification ×200). The tissue contained fibrous exudate and many neutrophils

A 28-year-old Chinese man had consumed a large amount of barbecued food and alcohol 7 days before admission to our hospital. He had felt abdominal pain after waking the next day, mainly in the upper abdomen. The position of the pain could not be described, and he gradually felt better without special treatment. He had had fever, abdominal pain, and pharyngeal pain 3 days before hospitalization, with his highest temperature reaching 41 °C. Our patient’s temperature then dropped after anti-infection treatment at his local clinic. One day before admission, his abdominal pain and fever reoccurred, mainly in the lower xiphoid process and upper abdomen. The abdominal pain, which was intermittent lacerating pain accompanied by back pain, was persistent and could not be relieved. He had nausea and vomiting, and the vomitus was the stomach contents. Our patient was then transferred to our hospital, and a physical examination revealed a body temperature of 38 °C, heart rate of 100 beats per minute, mild tenderness in the upper abdomen, mainly in the lower xiphoid process and left abdomen, no rebound pain, negative Murphy’s sign, and no pain on percussion in the liver and kidney areas. Laboratory data are shown in Table 1. A CT scan without contrast (Fig. 1a) showed an irregular soft tissue mass near the pancreatic head in the retroperitoneal space. The lesion was uneven in the interior and surrounded by a blurred fat gap. There were multiple spots of high density with clear margins in the upper right of the lesion. Contrast-enhanced CT scans (Fig. 1b-c) showed multilocular changes of the lesion, uneven enhancement of the cystic wall, slight enhancement of the adjacent duodenal wall, and multiple enlarged lymph nodes around the cyst wall. Gastroscopy (Fig. 2) revealed that the antral mucosa was rough and red and white in color with scattered patchy erythema. There was a deep fistula, about 0.3 cm in size, in the anterior wall of the duodenal bulb that was exuding white pus, and congestion and edema of the surrounding mucosa. A small amount of tissue around the fistula was removed, and pathological examination showed the tissue contained fibrous exudate and many neutrophils (Fig. 3). Subsequently, with the consent of our patient’s family members, an endoscopic anastomosis clip system (OTSC) of the duodenal fistula was successfully performed. After the operation, an enteral nutrition tube was inserted, and nasal feeding provided. Empiric anti-infection, acid-inhibiting, stomach- protecting, and symptomatic supportive treatments were given. Our patient’s body temperature gradually returned to normal and fluctuated within the normal range. No abdominal pain, abdominal distension, nausea, or vomiting reoccurred, and urine and stool were normal after a prescribed diet. Our patient was observed to reach a stable condition. One week and 1 month after treatment, abdominal CT (Fig. 1d) was reviewed and showed the volume of the lesion had gradually reduced and the edge was clear; however, there was no substantial change in the multiple high-density shadow spots on the upper right of the lesion.

**Table 1:** Laboratory data on admission

Project	Value	Project	Value
WBC	24.06 10 ^ 9/L	CA19–9	0.75 U/mL
RBC	5.34 10 ^ 12/L	Lipase	44.1 U/L
Neutrophil absolute value	18.81 10 ^ 9/L	Amylase	23.2 U/L
AFP	2.17 ng/mL	Anti-chain O	539.2 IU/mL
CEA	1.33 ng/mL	C-reactive protein	165 mg/L
Antinuclear antibody	Negative	Anti-tuberculosis antibody	Negative

*AFP* Alpha fetoprotein, *CA19–9* Glycoconjugate antigen 19–9, *CEA* Carcinoembryonic antigen, *RBC* Red blood cell, *WBC* White blood cell

## Discussion

A retroperitoneal abscess is a relatively uncommon clinical disease, the main causes of which are pancreatitis, appendicitis, perforations of the colon and rectum [6], and complications after surgery [7]. Duodenal perforation is also a relatively uncommon disease usually caused by diverticulum, infectious diseases (such as *H. pylori* infection), Crohn’s disease, enteritis caused by drugs or radiation, foreign bodies (such as fish bones), and trauma [2–4]. This case may have been caused by a lesion of the duodenum and enteritis due to overeating and drinking. In addition, the composition of barbecue meat is complex and may contain hard, sharp, and slender particles, which could increase the risk of duodenal perforation [2].

Nevertheless, retroperitoneal abscesses due to duodenal perforation are rare. A total of 13 cases of retroperitoneal abscesses caused by duodenal perforation have been reported in nine articles [8–16] from 1966 to 2018. In most cases, the patients were over 40 years old, with an average age of 60.3; in this case, our patient was 28 years old, and thus relatively young. The locations of duodenal perforations included the duodenal bulb (three cases) [8, 14, 16], the second segment of the duodenum (two cases) [13, 14], the third segment of the duodenum (two cases) [9, 15], and undetermined sites (six cases) [9–12]. In this case, the perforation was located in the anterior wall of the duodenal bulb, so the abscess was near the head of the pancreas. The main clinical symptoms of patients in all cases were abdominal pain, mainly in the right upper abdomen, occasionally accompanied by fever, nausea, and vomiting. In this case, fever was evident and abdominal pain was relatively mild. The cause may have been our patient’s forced position after drunkenness, which resulted in large amounts of food, alcohol, and digestive juices entering the retroperitoneal space through the duodenal fistula. Severe local inflammation was probably caused by a large number of foreign bodies.

Abdominal CT should be the first choice in the diagnosis of a retroperitoneal abscess. In the 13 reported cases, patients in the earlier stages did not undergo CT examination due to condition limitations, and only four patients underwent CT examination. The lesions of the four cases were all located in the retroperitoneum, they were solid lesions or cystic-solid lesions with blurred margins, and there was gas in some lesions. In this case, the location of the lesion was near the head of the pancreas because the perforation was located at the duodenal bulb. It was a cystic-solid lesion with punctate high-density shadows. Because of the interference of the punctate high-density shadows, the lesion was initially diagnosed as a mixed infection of tuberculosis and inflammatory exudation. The high-density shadows may have formed because the foreign body, which contained bone or other high-density substances, entered the retroperitoneal space through the duodenal fistula and was partially encapsulated by inflammatory tissue to form a retroperitoneal abscess.

Studies [8–16] show that surgery is the most common treatment for retroperitoneal abscesses caused by duodenal perforation. The mortality rate of patients with retroperitoneal abscesses by duodenal fistula was higher in the early stage because the retroperitoneal abscess was not found in time without a CT scan, and nursing and drainage technology were relatively poor. The main causes of death are sepsis, pneumonia, and pulmonary embolism. The mortality rate decreases significantly in the later stages due to the wide application of CT, improvements in drainage technology, the use of broad-spectrum antibiotics, and improvements in nursing methods. The endoscopic anastomotic clip system (OTSC), which has been proven to be effective for duodenal perforations and results in few complications [17], was used for the treatment of the duodenal fistula in this case. After surgery, a small intestinal nutrition tube was inserted and nasal feeding was provided with empiric anti-infection, acid-suppressing, stomach-protecting, and symptomatic supportive treatment. Our patient’s condition improved significantly. One month later, the volume of the retroperitoneal abscess was found to have decreased significantly. Compared with traditional surgical treatment, OTSC treatment causes less injury to patients and leads to faster recovery after surgery. OTSC treatment is suitable for patients with small fistulas, mild retroperitoneal abscesses, and good overall health.

## Conclusions

A retroperitoneal abscess caused by duodenal fistula is a rare disease. Abdominal CT is the preferred examination method. The abscess can be diagnosed by typical clinical symptoms and imaging results, and the appropriate treatment should be chosen based on the patient’s condition and sufficient imaging data.

## Abbreviations

AFP: Alpha fetoprotein
CA19–9: Glycoconjugate antigen 19–9
CEA: Carcinoembryonic antigen
CT: Computed tomography
OTSC: Endoscopic anastomosis clip system
RBC: Red blood cell
WBC: White blood cell
